# Latest advances in aging research and drug discovery

**DOI:** 10.18632/aging.102487

**Published:** 2019-11-21

**Authors:** Daniela Bakula, Andrea Ablasser, Adriano Aguzzi, Adam Antebi, Nir Barzilai, Martin-Immanuel Bittner, Martin Borch Jensen, Cornelis F. Calkhoven, Danica Chen, Aubrey D.N.J. de Grey, Jerome N. Feige, Anastasia Georgievskaya, Vadim N. Gladyshev, Tyler Golato, Andrei V. Gudkov, Thorsten Hoppe, Matt Kaeberlein, Pekka Katajisto, Brian K. Kennedy, Unmesh Lal, Ana Martin-Villalba, Alexey A. Moskalev, Ivan Ozerov, Michael A. Petr, Reason, David C. Rubinsztein, Alexander Tyshkovskiy, Quentin Vanhaelen, Alex Zhavoronkov, Morten Scheibye-Knudsen

**Affiliations:** 1 Center for Healthy Aging, Department of Cellular and Molecular Medicine, University of Copenhagen, Copenhagen, Denmark; 2 Global Health Institute, Ecole Polytechnique Fédérale de Lausanne (EPFL), Lausanne, Switzerland; 3 Institute of Neuropathology, University of Zurich, Zurich, Switzerland; 4 Max Planck Institute for Biology of Ageing, Cologne, Germany; 5 Cologne Excellence Cluster on Cellular Stress Responses in Aging-Associated Diseases (CECAD), University of Cologne, Cologne, Germany; 6 Department of Medicine, Institute for Aging Research, Albert Einstein College of Medicine, Bronx, NY 10461, USA; 7 Arctoris, Oxford, UK; 8 Gordian Biotechnology, San Francisco, CA 94107, USA; 9 European Research Institute for the Biology of Ageing (ERIBA), University Medical Center Groningen, University of Groningen, AD Groningen, The Netherlands; 10 Program in Metabolic Biology, Nutritional Sciences and Toxicology, University of California, Berkeley, CA 94720, USA; 11 SENS Research Foundation, Mountain View, CA 94041, USA; 12 Nestlé Research, EPFL Innovation Park, Lausanne, Switzerland; 13 School of Life Sciences, Ecole Polytechnique Fédérale de Lausanne (EPFL), Lausanne, Switzerland; 14 Haut.AI, Tallinn, Estonia; 15 Division of Genetics, Department of Medicine, Brigham and Women's Hospital, Harvard Medical School, Boston, MA 02115, USA; 16 Molecule, Basel, Switzerland; 17 Roswell Park Comprehensive Cancer Center and Genome Protection, Inc., Buffalo, NY 14203, USA; 18 Institute for Genetics and CECAD Research Center, University of Cologne, Cologne, Germany; 19 Department of Pathology, School of Medicine, University of Washington, Seattle, WA 98195, USA; 20 Department of Genome Sciences, University of Washington, Seattle, WA 98195, USA; 21 Department of Biosciences and Nutrition, Center for Innovative Medicine, Karolinska Institutet, Huddinge, Sweden; 22 Institute of Biotechnology, University of Helsinki, Helsinki, Finland; 23 Departments of Biochemistry and Physiology, Yong Loo Lin School of Medicine, National University Singapore, Singapore; 24 Centre for Healthy Ageing, National University Healthy System, Singapore; 25 Buck Institute for Research on Aging, Novato, CA 94945, USA; 26 Frost and Sullivan, Frankfurt am Main, Germany; 27 Molecular Neurobiology, German Cancer Research Center, Heidelberg, Germany; 28 Engelhardt Institute of Molecular Biology, Russian Academy of Sciences, Moscow, Russia; 29 Institute of Biology of Komi Science Center of Ural Branch of RAS, Syktyvkar, Russia; 30 Moscow Institute of Physics and Technology, Dolgoprudny, Russia; 31 Pharmaceutical Artificial Intelligence Department, Insilico Medicine, Inc., Rockville, MD 20850, USA; 32 Repair Biotechnologies, Inc., Syracuse, NY 13210, USA; 33 Department of Medical Genetics, Cambridge Institute for Medical Research, The Keith Peters Building, Cambridge CB2 0XY, UK; 34 UK Dementia Research Institute, The Keith Peters Building, Cambridge Biomedical Campus, Cambridge, UK; 35 Belozersky Institute of Physico-Chemical Biology, Moscow State University, Moscow, Russia

**Keywords:** aging, drug discovery, artificial intelligence

## Abstract

An increasing aging population poses a significant challenge to societies worldwide. A better understanding of the molecular, cellular, organ, tissue, physiological, psychological, and even sociological changes that occur with aging is needed in order to treat age-associated diseases. The field of aging research is rapidly expanding with multiple advances transpiring in many previously disconnected areas. Several major pharmaceutical, biotechnology, and consumer companies made aging research a priority and are building internal expertise, integrating aging research into traditional business models and exploring new go-to-market strategies. Many of these efforts are spearheaded by the latest advances in artificial intelligence, namely deep learning, including generative and reinforcement learning. To facilitate these trends, the Center for Healthy Aging at the University of Copenhagen and Insilico Medicine are building a community of Key Opinion Leaders (KOLs) in these areas and launched the annual conference series titled “Aging Research and Drug Discovery (ARDD)” held in the capital of the pharmaceutical industry, Basel, Switzerland (www.agingpharma.org). This ARDD collection contains summaries from the 6^th^ annual meeting that explored aging mechanisms and new interventions in age-associated diseases. The 7^th^ annual ARDD exhibition will transpire 2^nd^-4^th^ of September, 2020, in Basel.

## INTRODUCTION

Aging poses profound health-related challenges that need to be tackled to reduce the social and economic burden on our aging society. Multidisciplinary perspectives will be of tremendous importance to understand the underlying molecular processes of aging and to accelerate the discovery and development of effective aging interventions. It is therefore indispensable that industry and academia develop deeper cooperation and greater interchange of knowledge and technology. For this purpose, world leading experts from diverse research fields and various sectors came together at the 6th installment of the Aging, Drug Discovery and Artificial Intelligence conference, which was held from the 10^th^ to the 12^th^ September 2019 in Basel as part of the Basel Life Science Week. The event was co-organized by the teams lead by Morten Scheibye-Knudsen, Center for Healthy Aging, University of Copenhagen, Denmark, and Alex Zhavoronkov, Insilico Medicine, Hong-Kong. In the following, we provide an overview of the discussed research topics. The meeting followed on the heels of the 5^th^ ARDD [1].

### Challenges in aging research

Although great progress has been made towards the understanding of aging mechanisms, effective drug interventions are still missing for most age-related disorders. Targeting the aging process contrasts the traditional approach of “one disease-one drug”; thus, multiple challenges need to be overcome, as discussed by **Nir Barzilai** from the Albert Einstein College of Medicine, NY, USA. In particular, the political attention needs to be further strengthened by highlighting the clinical and economic benefits of aging interventions [2]. However, no party will cover intervention costs without an indication for which simple and reliable biomarkers are still lacking. Towards a resolution of this issue, the “Targeting Aging with Metformin” (TAME) study driven by Nir Barzilai may represent a proof-of-concept that could pave the way to clinical trials leading to healthy aging [3]. Indeed, metformin has been reported to extend lifespan in animal studies [4, 5] and linked to longevity in a retrospective clinical study on type 2 diabetes treatments [6]. A recently published human clinical trial further underlined its widespread effect based on transcriptomic data revealing gene expression changes of aging-linked metabolic and non-metabolic pathways [7].

Besides Metformin there are multiple other well-studied drugs that show high potential as aging drugs, as outlined by **Brian Kennedy** from the National University of Singapore, Singapore. He summarized known lifestyle interventions and small molecules that have been shown to cause lifespan extension. However, whether these aging interventions improve the organismal healthspan often remains a mystery. His research group recently revealed that the tricarboxylic acid cycle intermediate α-ketoglutarate increases the lifespan of mice [8]. Strikingly, the effect of α-ketoglutarate was even more profound on the healthspan than on the lifespan in mice that received the diet from the age of 18 months. Improved healthspan was indicated by a decreased level of inflammatory factors as well as a decreased frailty index.

But how can we fill the gap between lab animal research, which has traditionally stopped at murine studies, and human clinical trials? **Matt Kaeberlein** from the University of Washington, Seattle, USA, and colleagues several years ago initialized the dog aging project to overcome this barrier [9]. Companion animals like dogs as model organisms provide multifarious advantages including a faster aging pace than humans, high genetic diversity and a shared environment with humans [10]. The dog aging project aims to investigate the influence of genetic and environmental determinants on the life- and healthspan of domestic dogs based on survey, sequencing, blood biochemistry and -omics data collection. Further, the project provides the opportunity to test aging interventions, as already initiated for the mammalian Target Of Rapamycin (mTOR) inhibitor rapamycin. Notably, the completed phase 1 for the rapamycin intervention trial revealed no-side effects and improved cardiac function in treated dogs [11].

**Aubrey de Grey** from the SENS Research Foundation, Mountain View, California, USA, emphasized that placing the focus on healthspan and not on lifespan will help to rebut societal concerns for longevity investigations [12]. Further, he discussed that human diseases with a higher prevalence at older ages should be treated and explored differentially than communicable diseases. In this regard, he introduced the SENS Research Foundation (SRF) and their concept of maintenance by targeting mechanisms that mitigate cellular damage accumulating during aging. Notably, treatments of age-related diseases directed by spinouts of SRF aim to increase the healthspan of elderly - increased longevity is considered as a positive side-effect.

### Cellular pathways of lifespan regulation

Aging entails a functional decline of multiple cellular pathways that are required to maintain the cellular homeostasis. Nine hallmarks of aging were classified several years ago [13]; however, how these cellular mechanisms are regulated and interconnected is still not well understood. **Adam Antebi** from the Max Planck Institute for the Biology of Aging, Cologne, Germany, gave insights into how the nucleolus functions as a cellular stress signaling hub during aging [14]. Recent work from his group revealed a correlation between reduced nucleolar size, reduced nucleolar fibrillarin expression and extended lifespan in the nematode *C. elegans* [15]. In line with their observation, a *ncl-1* mutant strain with enlarged nucleoli reduced the lifespan of various genetic longevity models; conversely knockdown of nucleolar fibrillarin reduced nucleolar size and extended lifespan. Alongside reduced nucleolar size, long-lived *C. elegans* genotypes showed decreased ribosome biogenesis. A similar correlation between the nucleolus size and lifespan was observed in Drosophila, mice as well as in isolated muscle cells from elderly people who underwent a short-term period of reduced caloric intake and exercise. A function of the nucleolus in innate immunity was further demonstrated by the observation of decreased nucleolar size and fibrillarin expression upon bacterial infections [16]. These observations further link proteostasis and immune function with the aging process.

**Thorsten Hoppe** from the University of Cologne, Cologne, Germany, presented his latest research results regarding microRNA-dependent regulation of proteostasis and longevity. Recently, his research group used an *in vivo* reporter assay [17] to identify protein degradation defects in *C. elegans* mutants lacking the microRNA *mir-71* [18]. The study revealed that the microRNA *mir-71* regulates food odor perception and subsequently controls the expression of *tir-1* mRNA in AWC (amphid wing cell C) olfactory neurons. Disturbance of this pathway leads to reduced proteostasis in the intestine and premature aging in worms. Overall, their study highlighted a signaling axis between the brain and gut in response to food odor, a mechanism that may be relevant for age-associated neurological disorders such as Parkinson’s disease.

**Cornelis F. Calkhoven** from the European Research Institute the Biology of Ageing, Groningen, Netherlands, shared with the audience his latest research of the mTORC1 driven transcription factor C/EBPβ-LIP. Research of Cornelis Calkhovens group revealed that reduced expression of C/EBPβ-LIP in a mouse model decreased age-dependent physical decline, immune aging, and tumor incidence, while improving metabolic function [19, 20]. Notably, increased lifespan was observed in female but not in male mice [20]. Further, they used a compound library of FDA approved drugs in a luciferase-based reporter assay to identify drugs that reduce LIP expression [21]. In the long-term, pharmacological inhibition of LIP expression could be used to mimic the effects of caloric restriction.

### Aged stem cell rejuvenation

Stem cell rejuvenation holds great promise for the treatment of age-related disorders since stem cell exhaustion is thought to be a common feature of the aging process in multiple tissues [22]. Allowing self-renewal may boost the repair capacity of tissues and counteract the functional decline during aging. **Danica Chen** from the University of California, Berkeley, USA highlighted the Sirtuins, a NAD+-dependent protein deacetylase protein family, as key players during the aging process and as a promising target for stem cell rejuvenation [23]. Sirtuins were originally identified to increase the lifespan of yeast and the mammalian homologues Sirt2, Sirt3, and Sirt7 have been shown to be downregulated in aged hematopoietic stem cells (HSC) [24, 25]. Danica and her team uncovered a mitochondrial metabolic checkpoint guarded by sirtuins that ensures mitochondrial health in HSCs [26–28]. The mitochondrial metabolic checkpoint becomes dysregulated in HSCs of old mice, resulting in loss of HSC maintenance due to NLRP3 inflammasome activation. Notably, targeting the Sirtuins-NLRP3 signaling pathway improved the function and repair capacity of aged HSCs.

**Ana Martin-Villalba** from the German Cancer Research Center, Heidelberg, Germany, introduced the audience to the world of neuronal stem cell regeneration. With age, the number of neuronal stem cells (NSCs) declines in the subventricular zone. Recently, Ana Martin-Vallalbas group revealed that a fast decline of all subpopulations of NSCs occurs from young to middle-aged mice, however, in old mice the decline was slowed down [29]. The remaining population of neuronal stem cells is maintained in a resistant quiescent state by inflammatory signals. However, once activated old NSCs show a functional similarity to young NSCs. Highlighting the potential of NSCs re-activation in the aged brain to counteract age-related neurological decline.

**Jerome Feige** from Nestlé Research, Lausanne, Switzerland, emphasized different targeting strategies for augmenting the repair capacity of aged muscle stem cells. Loss of muscle mass and function can start at the early adult stage and lead to sarcopenia, which contributes vastly to the diminished life quality of the elderly [30]. The integrity of muscle stem cells is not only influenced by intrinsic mechanisms but also by the diverse muscle stem cell microenvironment, including fibro-adipogenic progenitor cells (FAPs) [31]. In line with this, recent work from his group revealed targeting of the muscle stem cell niche as a promising intervention strategy [32]. Transcriptome profiling identified the FAPs secreted matricellular protein WISP1 as an important factor for maintaining the integrity of muscle stem cells. Consequently, the expression of WISP1 declines during age and restoration of WISP1 expression counteracts the loss of muscle regeneration capacity.

Targeting the stem cell niche to rejuvenate stem cell function was also discussed by **Pekka Katajisto** from the Institute of Biotechnology, HiLIFE, University of Helsinki, Helsinki, Finland and the Karolinska Institute. Intestinal stem cells (ISCs) are supported by Paneth cells that are specialized epithelial cells localized in the stem cell niche. Caloric restriction inhibits mTORC1 signaling in Paneth cells, which promote ISC function via paracrine mechanism, and thereby improve regenerative capacity of intestinal epithelium after irradiation in mice [33]. During aging, the functionality of ISCs and Paneth cells decreases in human and in mice [34]. The loss of function is in part caused by an increase in Notum secreted by Paneth cells. Notum is a deacylase that inactivates Wnt ligands necessary for ISC maintenance and function. Strikingly, the Notum inhibitor ABC99 increased the Wnt signaling pathway in ISCs and restored the functionality of aged ISCs *in vivo*.

### Drug discovery in aging research

Different strategies are pursued for the identification of aging interventions: *de novo* drug discovery or repurposing of existing drugs. In particular, repurposing of FDA-approved drugs provides numerous advantages, including lower costs and shorter timeline for the drug development pipeline. **Alexey Moskalev** from the Moscow Institute of Physics and Technology, Moscow, Russia, approached the question if aging drug discovery is becoming a reality. Geroprotection, is not a recent idea and already received attention in the 1950s where Denham Harman proposed the free radical theory of aging and the potential of antioxidants [35]. Since that time, more than 250 compounds have been shown to increase lifespan in aging model organisms by targeting cellular processes such as autophagy, cellular senescence or DNA repair [36]. As aging is a multifaceted process, recent studies indicate that a combined use of drugs leading to healthy aging may increase the benefit of single interventions [37, 38]. However, clinical trials for geroprotectors are still lacking due to the missing availability of reliable biomarkers and beneficial drug classification [39].

One of the first and best-studied aging targets is the nutrient sensor mTOR. However, the regulatory mechanism is still not fully understood and studying mTOR regulation may help to identify new interventions in the future, as outlined by **David Rubinsztein** from the Cambridge Institute of Medical Research, University of Cambridge, UK. Recent work by David Rubinsztein’s group highlights the regulatory mechanism that leucine imposes on mTOR signaling [40]. Interestingly, in most of the cell types they studied, the described signaling pathway was driven by the leucine metabolite acetyl-coenzyme A and was independent of any, so far, identified leucine receptor. However, leucine sensing does appear to be mediated by leucine sensors in some cell types, like HEK293 cells. In addition, David Rubinsztein underscored the potential of drug repurposing for the identification of autophagy inducers in brain disease [41]. A screen for FDA-approved L-type calcium channel blockers identified Felodipine as a strong autophagy inducer. Felodipine showed neuroprotective effects in a mouse model of Parkinson’s disease at plasma concentrations similar to those seen in people taking the drug for hypertension.

**Anne Bertolotti** from the MRC, Cambridge, UK, discussed her research concerning the selective inhibition of phosphatases to enhance the protein quality control system in neurological disorders. Guanabenz was identified to selectively inhibit the protein phosphatase PPP1R15A, thereby inhibiting the dephosphorylation of elF2alpha [42]. Prolonged activation of elF2alpha reduced ER-stress caused by misfolded proteins via regulating protein translation/chaperone availability rate. Based on the promising effects of Guanabenz, its derivate Sephin1 prevented molecular and physiological changes in disease models of Charcot-Marie-Tooth 1B and amyotrophic lateral sclerosis [43]. To expand the approach of phosphatase inhibition, Anne Bertolottis group developed a platform to screen for phosphatase inhibitors [44]. As a proof-of-concept, Raphin1 was identified as novel PPP1R15B inhibitor that attenuates neurological decline in a Huntington’s disease mouse model.

**Andrea Ablasser** from the Global Health Institute, École Polytechnique Fédérale de Lausanne (EPFL), Lausanne, Switzerland, discussed the potential of targeting the cytosolic DNA sensing pathway cGAS-Sting in human disease [45, 46]. The cGAS-Sting pathway triggers the inflammatory response and thus it represents a promising target for inflammatory-driven diseases. Recently, her group identified C-176, C-178 and its derivates as small-molecule inhibitors of STING mediated IFNbeta response [47]. A three-month treatment with C-176 in a mouse model of inflammatory disease strongly reduced inflammatory parameters. Targeting the cGAS-Sting pathway may also be relevant for senescence-mediated pathologies as the cGAS-Sting pathway was recently shown to be a crucial regulator of cellular senescence [48].

**Morten Scheibye-Knudsen** from the Center for Healthy Aging, Copenhagen, Denmark, highlighted the diversity of aging features consistent with the complexity of the aging process [49]. He supplied evidence for a role of DNA damage in the aging process both through highlighting that loss of DNA repair leads to premature aging and by showing novel data suggesting that stimulating DNA repair might significantly extend the lifespan of model organisms. Importantly, in collaboration with Insilico Medicine, his team has discovered an abundance of small molecules able to stimulate DNA repair.

**Andrei Gudkov** from the Department of Cell Stress Biology at Roswell Park Comprehensive Cancer Center, and its spinoff biotech company Genome Protection, Inc. (GPI), both in Buffalo, NY, discussed his ongoing work regarding the impact of genotoxic stress as a driver of cellular senescence and aging. Recently, a research study indicated that the re-activation of retro transposable elements may be a source of DNA damage during aging [50]. Inhibition of LINE1-encoded reverse transcriptase significantly reduced age-related systemic inflammation, accumulation of DNA damage markers and may provide a target for antiaging and anticancer interventions. Andrei Gudkov also briefly discussed the hurdle to assess the health status of organisms such as mice. In order to simplify this, they developed the *physiological frailty index*, a non-invasive method to determine the biological age of mice [51].

**Quentin Vanhaelen**, from Insilico Medicine, shared their recent work showing how AI for generative chemistry can be used to drive rapid drug discovery. Point in question was the demonstration that efficacious drugs can be developed in just 21 days for a new target. Here, an effective inhibitor of discoidin domain receptor 1 (DDR1), a kinase target implicated in fibrosis, was generated [52]. Clearly, AI driven discovery has now progressed to a level where small molecule design can be done more rapidly and accurately than ever before. Generative chemistry technology combined with other computational chemistry techniques was applied to develop specific and selective modulators for multiple targets implicated in aging and age-related diseases.

**Adriano Aguzzi** from the Institute of Neuropathology, University of Zürich, Zürich, Switzerland, discussed the latest development in the research of prion diseases. Prion diseases are a group of neurodegenerative disorders that are caused by misfolding and aggregation of the prion protein PrPC [53]. Adriano Aguzzi shared his recent results regarding the disease-causing mechanism and possible interventions. Notably, prion diseases share some clinical and molecular features with age-related neurodegenerative diseases such as Alzheimer’s disease and Parkinson’s disease [54]. Thus, new insights in one of them may help to find interventions for all of these devastating disorders.

### Big data analysis and technologies to accelerate aging drug discovery

Recent advances in omics technologies have dramatically increased the volume of data representing the complexity of cellular processes and diseases. But how can we implement big data to increase our knowledge about aging processes and aging drug discovery? **Vadim Gladyshev** from Brigham and Women’s Hospital, Harvard Medical School, MA, USA highlighted the importance of discovering novel biomarkers of aging to assess the effect of longevity interventions in humans and animal models. His group identified longevity signatures based on comparative analysis of transcriptomic and metabolomic data. Signatures were identified by comparing short-lived and long-lived species [55, 56], interventions leading to longevity [57], different ages [58] and different cell types [59]. These signatures could be used to identify novel longevity interventions.

**Alexander Tyshkovskiy** from Brigham and Women’s Hospital, Harvard Medical School, Boston, MA, USA, and Belozersky Institute of Physico-Chemical Biology, Moscow State University, Moscow, Russia, gave insights into the gene expression signatures of different longevity interventions in mice. To identify common patterns of different interventions, they analyzed transcriptomic data of mice of both sexes and different ages subjected to eight different longevity interventions [57]. Combining their data with published data from other groups, they identified gene expression signatures associated with lifespan extension. Notably, they developed the web-based tool GENtervention (http://gladyshevlab.org/GENtervention/), which can be utilized to investigate associations between genes and longevity. Overall, the identified signatures may help to identify new lifespan-extending interventions in the future.

Clearly, the analysis of large datasets has become an essential part of biomedical research where Insilico Medicine is a front-runner on the industrial side. In this meeting, **Ivan Ozerov**, from Insilico Medicine, introduced the multi-omics and drug discovery pipeline Pandomics [60]. He demonstrated how published gene-expression datasets can be combined with user owned unpublished data, and how that can lead to the identification of altered pathways as well as possible molecules. Notably, the toolset gives information of possible patents and other information that could guide industries in decision making regarding the pursuit of specific small molecules.

**Michael A. Petr** from the Center for Healthy Aging, University of Copenhagen, Copenhagen, Denmark, emphasized the advantages of studying aging processes and interventions in *M. musculus* and *D. melanogaster* animal models*.* Here he presented a specific case of studying various model organisms of a premature aging disorder in a single study, applying an intervention to the models, and developing a technology to automate the phenotyping process. The technology evolved due to the limitations of high time and labor demand, as well as costs to phenotype any model organism. Thus, he and his colleagues developed a system utilizing computer vision and deep learning to finely track model organisms allowing hundreds of conditions to be tested in parallel, while simultaneously generating a comprehensive palette of data outputs (unpublished data, http://www.tracked.bio).

### The longevity industry

In addition to the academic talks, several companies presented solutions to multiple issues within the aging and drug discovery field. Martin **Borch Jensen**, from Gordian Biotechnology, San Francisco, USA, presented a discovery platform that allows simultaneously screening thousands of therapeutics in individual animal models. This addresses the problem that age-related diseases involve a complex interplay between cells and the aged environment, which is not captured with traditional screening methods. By starting the drug discovery process with high-throughput target validation in realistic, aged, disease environments, the time and cost of later development for inefficacious targets can be avoided.

A major obstacle to drug development lies with the initial conversion of academic ideas to fundable small molecules. Here, **Tyler Golato**, from Molecule Protocols, Basel, Swezerland, demonstrated their distributed IP platform whereby multiple investors can spread the risk of small molecule development by sharing initial early investment. This platform may solve a major problem in drug development for academia where the jump to commercialization is often very difficult.

**Reason**, from Repair Biotechnologies, Syracuse, NY, USA, introduced the two ongoing projects of the newly established company Repair Biotechnologies. The projects focus on gene therapy-based repair of the two common aging features thymic atrophy and atherosclerosis that cause immunological dysfunction and cardiovascular disease. Recently, Repair Biotechnology has closed their first funding round allowing them to test their therapies in animal models with the long-term goal for clinical transition.

**Anastasia Georgievskaya**, from Haut.Ai, Tallinn, Estonia, demonstrated how AI and computer vision technology can be implemented to develop powerful tools to study skin pathologies. Recently, Haut.AI developed the PhotoAgeClock, a non-invasive biomarker that can predict the age of humans with an accuracy of 2.3 years mean absolute error [61].

**Martin-Immanuel Bittner**, from Arctoris Ltd, Oxford, UK, showed us how drug discovery and development can be accelerated by making use of automation. One of the applications of automation and robotics is in characterizing novel compounds such as senolytic agents applicable for targeting age-related diseases. He made the argument that automated experimentation using robotics is the perfect companion to next generation AI-driven drug discovery, by providing large amounts of structured, validated, and highly reproducible data.

**Unmesh Lal**, from Frost and Sullivan, ended the meeting with an overview of the aging, drug discovery and AI field. He highlighted the many industry partners involved and the opportunities for growth.
